# Associations between Total and Added Sugar Intake and Diabetes among Chinese Adults: The Role of Body Mass Index

**DOI:** 10.3390/nu15143274

**Published:** 2023-07-24

**Authors:** Yan Liu, Jing Cheng, Lijin Wan, Wei Chen

**Affiliations:** 1 Department of Clinical Nutrition, Peking Union Medical College Hospital, Chinese Academy of Medical Sciences, Beijing 100730, China; shyneeliu@163.com; 2 Division of Oral Epidemiology & Dental Public Health, University of California at San Francisco, San Francisco, CA 94143, USA; jing.cheng@ucsf.edu; 3 Baidu Inc., Beijing 100193, China; wanlijin@hotmail.com

**Keywords:** total sugar, added sugar, diabetes, body mass index, mediation analysis, adults

## Abstract

Sugar intake has been linked to the global rise in diabetes. However, the unique diabetogenic effect of sugar, independent of weight gain, remains controversial. This study aimed to investigate the associations between total and added sugar intake and diabetes status, and to test whether the sugar–diabetes associations were moderated or mediated by the body mass index (BMI). We performed a nationwide cross-sectional study on 12,889 Chinese adults who were enrolled in the China Health and Nutrition Survey (CHNS) 2011. The data for the total and added sugar intake were measured using three consecutive 24 h recalls, and determined based on the U.S. Department of Agriculture (USDA) National Nutrient Database for Standard Reference, Release 28 (SR28), the Food Patterns Equivalents Database (FPED) 2015–2016, and the labeled ingredients and nutrient contents. A multivariable logistic regression model was used to analyze the associations between the total and added sugar intake and diabetes. A nutrient density model was used to adjust for the total energy intake. A mediation analysis for significant sugar–diabetes associations shown in multivariable logistic analysis (*p* < 0.05), and a subgroup analysis according to the BMI category were performed, to examine the mediating and moderating effects of the BMI on the sugar–diabetes association, respectively. We included 12,800 individuals, with a mean age of 50.5, in the final analysis. The means of the total and added sugar intake, total sugar (%E), and added sugar (%E) were 28.2 ± 0.2 g/d, 5.0 ± 0.1 g/d, 6.0 ± 0.0%, and 1.0 ± 0.0%, respectively. The overall prevalence of self-reported physician-diagnosed diabetes was 4.0%. A significant association between the total sugar intake and an increased risk of diabetes was found (odds ratio [OR] =1.008, 95% CI 1.001–1.016). The mediation analysis showed a significant mediation effect through the BMI of the effect of the total sugar on diabetes status (*p* < 0.001), where 11.7% (95% CI: 4.7–35.7%) of the effect of the total sugar on diabetes was mediated through the BMI. The total sugar intake had a significant direct effect on diabetes around the BMI (estimated coefficient = 0.0004, *p* < 0.001). The overall total-sugar-intake–diabetes association remained significant in normal-weight participants in the subgroup analysis (OR =1.012, 1.000–1.024). In conclusion, although the BMI moderated and mediated the association between the total sugar intake and diabetes, the total sugar still showed some unique weight-independent diabetogenic effects. Our findings call for efforts to prevent and control diabetes by reducing sugar intake, and losing weight appropriately.

## 1. Introduction

Diabetes is an important global health issue affecting approximately 537 million adults aged 20–79 years, and imposing a substantial disease and economic burden on all countries [1,2,3]. Alarmingly, the epidemic has increased rapidly over recent decades, parallel to the prevalence of obesity [4,5], and is projected to reach 783 million by 2045 [1,3,6]. In addition, according to estimates, over 6.7 million adults aged 20–79 years will die from diabetes-related causes in 2021 [1]. Thus, identifying the etiology of diabetes and developing appropriate prevention strategies are critical.

The etiology of diabetes is believed to be multifactorial, and involves an interplay between genetic, dietary, physical activity, and environmental factors [4,7,8]. Of these, dietary sugars have been long purported to increase the risk of diabetes, with a particularly compelling biological plausibility. For instance, glucose and maltose cause a rapid rise in postprandial glycemia [9], which is an independent risk factor for the development of type 2 diabetes [10]. However, sugars such as sucrose and fructose have a medium-to-low glycemic index, and contribute to a small proportion of blood glucose [9]. Their diabetogenic effect cannot be explained by elevated blood glucose. As obese people tend to eat more sugar [11,12], the mechanism by which sugars cause insulin resistance through obesity, the strongest risk factor for developing diabetes [13,14], has raised enormous consideration. Epidemiological studies even suggest that sugars may not have a causal impact on diabetes, other than providing calories [15]. However, a large-scale prospective cohort study showed that after adjusting for the body mass index (BMI) and energy intake, sugar-sweetened soft drinks still play a deleterious role in diabetes [12]. The unique diabetogenic effect of sugars, independent of weight gain, remains controversial.

Therefore, this study aimed to use the China Health and Nutrition Survey (CHNS) 2011, a nationwide cross-sectional study with a representative sample of Chinese individuals and a standard design and strict quality control procedure, to investigate, for the first time in China, the associations between the total and added sugar intake and diabetes status, and to test whether the associations were mediated or moderated by the BMI.

## 2. Methods

### 2.1. Study Sample

The data for this study were derived from the 2011 wave of the CHNS, the most recent national dietary data available from the CHNS to date. The CHNS is a nationwide prospective cohort study conducted every 2–4 years since 1989 using a multi-stage, random clustering process to draw a sample of over 30,000 participants in China [16]. The sampling process involved five stages. In the first stage, 15 provinces in mainland China were chosen at random, including Beijing, Chongqing, Guangxi, Guizhou, Heilongjiang, Henan, Hubei, Hunan, Jiangsu, Liaoning, Shanghai, Shandong, Shaanxi, Yunnan, and Zhejiang. In the second stage, the sample for each province was chosen using a multi-stage, random cluster method. In the third stage, counties and cities in each province were stratified by income (low, medium, and high), and a weighted sampling scheme was used to randomly select four counties and two cities in each province. In the fourth stage, villages and townships within the counties and urban and suburban neighborhoods within the cities were randomly selected. In the final stage, 20 households were randomly selected in each community, and all household members were interviewed [17].

This study included adults aged 18 years or older who participated in the CHNS in 2011 with dietary information for two or more days (*n* = 12,889). Participants were excluded when no diabetes diagnosis information was available (*n* = 89), leaving 12,800 individuals. The CHNS was approved by the ethical review committees of the Chinese Center for Disease Control and Prevention, and the Carolina Population Center at the University of North Carolina at Chapel Hill. Signed, informed consent was obtained from all participants before the survey.

### 2.2. Assessment of Sugar Intake

The method of assessing sugar intake has been described previously, and is briefly outlined herein [18]. Individual dietary intakes were collected from three consecutive 24 h recalls. A household food inventory and an in-house weighing approach were used to check the quality of the 24 h recall data. The nutrient content of foods consumed each day, including the intake of total calories, carbohydrates, fats, and proteins, was determined by the CHNS, using the 2004 China Food Composition Tables (FCTs) [19]. As data on the total and added sugar content are not yet available in the FCTs, the total and added sugar intake was determined using the U.S. Department of Agriculture (USDA) National Nutrient Database for Standard Reference, Release 28 (SR28), and the Food Patterns Equivalents Database (FPED) 2015–2016 [20,21]. The amount of total and added sugars consumed in each food was determined by assigning values to items with exact food description matches, and imputing independent estimates for items without an exact match; this was carried out by two investigators (Y.L. and L.W.). Because data on the total and added sugar content are not available on food nutrition labels in China, processed foods that had no similar comparison food in the SR28 or FPED 2015–2016 dataset but were sold in Chinese supermarkets in San Francisco were estimated using the labeled ingredients and nutrient contents. To calculate the amount of total and added sugar in grams, teaspoon equivalents were multiplied by 4.2 g/teaspoon, and then multiplied by the total amount (in grams) of each food. The average daily intake of total and added sugar was then calculated for each participant. Finally, the results were multiplied by 4 kcal, and divided by the total energy intake (in kcal/d), to obtain the percentage of total energy (%E) from total or added sugar.

### 2.3. Assessments of Covariates

Self-reported demographics (e.g., age, gender) and lifestyle information (e.g., smoking status, drinking frequency, and physical activity) were obtained through a structured interview. The weight and height of participants were measured based on the WHO standard, using calibrated beam scales and portable stadiometers, respectively. The BMI was defined as weight in kilograms divided by height in meters squared. Overweight and obese were defined as a BMI of 24.0–27.9 kg/m^2^ and 28.0 kg/m^2^ or higher, respectively, according to the BMI cut-off values proposed by the Working Group on Obesity in China [22]. Physical activity was assessed based on four components, including occupational, household, transportation, and leisure activities. The estimates of the total physical activity levels were calculated by multiplying a specific energy expenditure in the metabolic equivalent tasks (METs) of each activity by the hours spent on the corresponding activity per week, and summing the values of all activities [23,24].

### 2.4. Definition of Diabetes

As the outcome, diabetes was identified by using the questionnaire-based interview. Self-reported physician-diagnosed diabetes was considered if participants answered ‘yes’ to the question: “Has a doctor ever told you that you suffer from diabetes”?

### 2.5. Statistical Analysis

The basic demographics and characteristics of the participants were summarized as means and standard errors (SEs) or medians and interquartile ranges (IQR) (25th, 75th) for the continuous variables (age, physical activity, dietary intake, etc.), and frequencies (percentages) for the categorical variables (age group, gender, BMI category, smoking status, drinking frequency, etc.), both overall, and by total sugar intake. Specifically, participants were divided into Group 1 (lower sugar intake) and Group 2 (higher sugar intake) according to the median value of the total sugar intake (21.9 g/d), and participants’ demographics and characteristics were summarized by the sugar intake groups. Student’s *t*-test, Mann–Whitney U test, or chi-squared test were used for the between-group comparisons, as appropriate. The total and added sugar intake, the percentage of total energy from total sugar (total sugar (%E)), and the percentage of total energy from added sugar (added sugar (%E)) were presented both as means with standard errors (SEs) and medians with IQR (25th, 75th). Comparisons on %E between the low and high sugar groups were performed using the Mann–Whitney U test. Given the large sample size of the study, we expected a statistically significant difference between the two groups in most characteristics, but would consider a difference of at least 5% as clinically meaningful.

In the nutrient density model, we used multivariable logistic regression models to analyze the associations between the total and added sugar intake per 1000 kcal and diabetes, while controlling for covariates. Specifically, the primary analysis was conducted using Model 1 to evaluate the sugar-intake–diabetes association, while controlling for basic demographics (age and gender); Model 2 was used to investigate the robustness of the sugar-intake–diabetes association found in Model 1, after further controlling for lifestyle variables (smoking status, drinking frequency, and physical activity) [25]. Given the evidence that the effect of sugar intake on diabetes may be influenced by the cumulative effects of obesity [15], mediation analysis was performed to investigate whether, and how much, sugar intake increased the odds of developing diabetes via and around the BMI, and subgroup analysis was performed via the BMI category (<24.0 kg/m^2^, 24.0~27.9 kg/m^2^, or ≥28 kg/m^2^) to explore potential heterogeneous associations between sugar intake and diabetes in the BMI subgroups [22]. The mediation analysis fitted a linear mediator model for the BMI on sugar intake per 1000 kcal, and a logistic outcome model for diabetes status on sugar intake per 1000 kcal and BMI. Age, gender, and other Model 2 covariates were included in the mediator and outcome models. The nonparametric bootstrap method with 500 repeats was used for the confidence intervals of parameters.

All tests were two-sided, and statistical significance was determined at *p* < 0.05 (two-tailed tests). The mediation analysis was conducted with R statistical package 4.2.2 (http://www.r-project.org (accessed on 21 January 2023)), using the “mediation” package. Other analyses were performed using International Business Machines (IBM) Statistical Package for the Social Sciences (SPSS) version 26.0 (IBM Corp., Armonk, NY, USA).

## 3. Results

### 3.1. Participant Basic Demographics and Characteristics by Total Sugar Intake Levels

Of all the included participants, more than half (52.9%) were female. The mean age was 50.5 years. The higher-sugar-intake group was significantly younger, and contained more males, overweight or obese people, non-smokers, and never-drinkers, and those with higher physical activity, total energy intake, total fat intake, total protein intake, and total carbohydrate intake than the lower-sugar-intake group (all *p* < 0.05). However, the magnitude of the differences in age and the distribution of age group, gender, and smoking status between the groups was not considered clinically meaningful (Table 1).

### 3.2. Total and Added Sugar Intake of Diabetics and Non-Diabetics by Age, Gender, and BMI Category

Our result showed that the overall prevalence of self-reported, physician-diagnosed diabetes was 4.0%. The total sugar intake, added sugar intake, total sugar (%E), and added sugar (%E) by age, gender, and BMI categories among participants with different diabetes statuses are presented in Table 2. The means ± SEs and medians (25th, 75th) of the total and added sugar intake, total sugar (%E), and added sugar (%E) of all the included participants were 28.2 ± 0.2 g/d [21.9 (10.7, 39.0) g/d], 5.0 ± 0.1 g/d [0.0 (0.0, 3.5) g/d], 6.0 ± 0.0% [5.0 (2.5, 8.4)%], and 1.0 ± 0.0% [0.0 (0.0, 0.8)%], respectively. The total and added sugar intake and added sugar (%E) were higher in diabetics aged 45–59 years than that in non-diabetics in the same age group (all *p* < 0.05). A similar phenomenon was observed for the total sugar intake among participants aged 60 years or older (*p* = 0.004). These four variables were higher in diabetic males than in non-diabetic males (all *p* < 0.01). In addition, normal-weight diabetics had a higher total sugar (%E) than non-diabetics in the same BMI category (*p* = 0.011). Overweight diabetics also had higher total sugar (%E), added sugar intake, and added sugar (%E) than overweight non-diabetics (all *p* < 0.05). All of the above differences between diabetics and non-diabetics were clinically meaningful.

### 3.3. Association between Total and Added Sugar Intake and Diabetes Status

In the multivariable logistic analysis on the association between the total and added sugar intake and diabetes, we found that a higher total sugar intake was significantly associated with higher odds of diabetes in Model 1 (adjusted for age and gender) and Model 2 (adjusted for age, gender, smoking status, drinking frequency, and physical activity). There were no associations between the added sugar intake and diabetes in either model (Table 3).

### 3.4. Mediating Effect of BMI on the Total-Sugar–Diabetes Association

**Figure 1:**
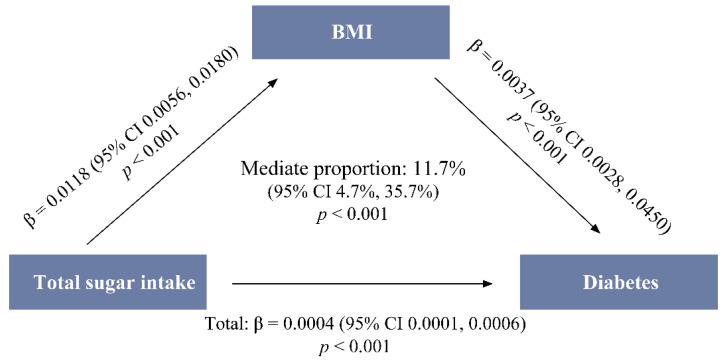
Mediation analysis between the total sugar intake and diabetes using the BMI ^1^, CHNS 2011. ^1^ BMI: body mass index; CHNS: China Health and Nutrition Survey.

The mediation analysis demonstrated that total sugar not only has a significant direct effect on diabetes around the BMI (estimated coefficient = 0.0004, *p* < 0.001), but also has a significant indirect effect on diabetes through the BMI, which mediated 11.7% (95% CI 4.7–35.7%, *p* < 0.001) of the total-sugar–diabetes association (Figure 1).

### 3.5. Moderating Effect of the BMI on the Sugar–Diabetes Association

In the subgroup analysis according to the BMI category, a significant association between the total sugar intake and diabetes was observed in participants with a BMI value < 24.0 kg/m^2^ in either the minimally adjusted or fully adjusted models (Table 4).

## 4. Discussion

In this nationwide cross-sectional study, with data collected from 12,800 adults in the CHNS 2011, we provide the first evidence of a significant association between total sugar intake and increased odds of diabetes in China, which remained significant only in normal-weight participants in the subgroup analysis, after adjusting for participants’ age, gender, and lifestyle. In addition, we found that although the BMI mediated 11.7% of the total-sugar–diabetes association, the total sugar intake still exhibits significant unique weight-independent diabetogenic effects.

Consistent with our study, the associations between sugar intake and diabetes have been previously reported by a number of studies. For instance, based on a large cohort of 38,480 initially healthy postmenopausal women followed for an average of 6 years, Janket, S. J. et al. reported a definitive effect of sugar intake on the risk of developing type 2 diabetes, after adjusting for age and smoking [26]. A systematic review and meta-analysis reported that a habitual consumption of sugar-sweetened beverages was associated with a greater incidence of type 2 diabetes [27]. However, both found that the BMI weakens or eliminates the association between sugar intake and diabetes, indicating that the effect of sugar intake on diabetes may be moderated by the cumulative effects of obesity, or that the extra calories from sugars may indirectly promote diabetes via obesity-mediated mechanisms.

To explore the moderating and mediating effect of the BMI on total-sugar–diabetes associations, we conducted a subgroup analysis according to the BMI status, and mediation analysis. Interestingly, in the subgroup analysis, we found that the total sugar intake was positively associated with diabetes in participants with a BMI value < 24.0 kg/m^2^, whereas no association was observed in those with higher BMI values, after controlling for participants’ age, gender, and lifestyle. Our findings in the normal-weight group suggest that the total sugar intake has some unique weight-independent diabetogenic effects. This speculation was confirmed by our mediation analysis of the pathway from the total sugar intake to the BMI to diabetes, which showed that the total sugar not only had an indirect effect on diabetes via the BMI, but also had a direct effect on diabetes around the BMI. This can be explained by some biological mechanisms. In addition to elevating the blood glucose [9], the sugar-mediated generation of uric acid can stimulate oxidative stress, endothelial dysfunction, and inflammation [28], which have been suggested as comprising a potential mechanism for insulin resistance [29,30]. With regard to the results of the overweight and obesity groups in the subgroup analysis, their total sugar (%E) was 6.2 ± 0.1% (5.3 [2.7, 8.6]) and 6.5 ± 0.1% (5.7 [2.9, 8.9]), respectively, far below the sugar intake limit (the free sugars or added sugars consumed making up less than 10% of the total energy intake per day) recommended by the World Health Organization (WHO) and the Dietary Guidelines for Americans (DGA) 2020–2025 [31,32]. The impact of the total sugar on diabetes may be overshadowed or blocked by high BMI values, the strongest risk factor for developing diabetes [13,14]. This also explains our findings that showed that no association existed between added sugar and diabetes.

With the largest number of adults aged 20–79 years with diabetes in 2021 [1], China requires a broad-based prevention strategy. Based on our findings, and the upward trends in the total and added sugar intake in China [18], a limit for sugar intake may be a promising approach for diabetes control, especially in those of normal weight. Thus, promoting the Dietary Guidelines for Chinese Residents (2022) that control the daily added sugar intake to no more than 50 g, and preferably less than 25 g; reducing the sugar content in reformulated processed foods; and improving correct cognition and healthy attitudes toward healthy food choices would be effective strategic priorities [33]. Furthermore, weight loss, achieved through various measures, such as lifestyle changes, diet, and increased physical activity, is crucial in preventing diabetes development in overweight and obese individuals [34,35].

To the best of our knowledge, this is the first study to provide data on the total and added sugar intake among Chinese adults. This is also the first to estimate the sugar–diabetes association in China, taking into account both the moderating and mediating effects of the BMI. The strengths of this study also include its standardized design, nationally representative sample size, and rigorous dietary-measurement procedures, allowing us to provide a relatively accurate report of the sugar–diabetes association in the largest developing country, with the largest number of diabetic adults, in the world.

Our study had several limitations. Firstly, as the data on the total and added sugar content are not yet available in the China Food Composition, the SR28 and FPED 2015–2016 combined for the labeled ingredients and nutrient contents were used, which may not fully apply to Chinese foods. Secondly, our study relied on self-reported physician-diagnosed diabetes. The prevalence of diabetes was likely underestimated. Thirdly, because the CHNS has not yet published dietary data after 2011, our study used data from the CHNS 2011, which are the most recent national dietary data available from the CHNS to date.

## 5. Conclusions

In conclusion, in this nationwide cross-sectional study of 12,800 participants, we report a significant association between total sugar intake and increased odds of diabetes, which remained significant in normal-weight participants in the subgroup analysis. In addition, we found a significant mediation effect of the total sugar, through the BMI, on diabetes status. The total sugar intake also had a significant direct effect on diabetes around the BMI. Taken together, our study provides further evidence that although the BMI moderated and mediated the association between the total sugar intake and diabetes, the total sugar still exhibits direct diabetogenic effects. These findings call for efforts to tackle diabetes by encouraging people to reduce their sugar intake and lose weight appropriately.

## Figures and Tables

**Table 1:** Basic characteristics of Chinese adults by total sugar intake, CHNS ^1^ 2011.

	Total (*n* = 12,800)	Binaries of Total Sugar Intake		
		Group 1 (Lower; *n* = 6400)	Group 2 (Higher; *n* = 6400)	*p*-Value
Characteristics [mean ± SE ^1^ or median (25th, 75th)]				
Age (year)	50.5 ± 0.1	51.4 ± 0.2	49.7 ± 0.2	<0.001
Physical activity (MET ^1^ h/week)	194.2 (44.8, 609.1)	148.6 (38.0, 612.3)	269.4 (52.5, 604.7)	<0.001
Total energy intake (kcal/d)	1830.9 (1444.9, 2291.8)	1668.8 (1305.3, 2133.1)	1975.9 (1596.9, 2429.3)	<0.001
Total fat intake (g/d)	65.9 (46.3, 90.5)	62.2 (43.3, 86.4)	69.8 (49.6, 93.9)	<0.001
Total protein intake (g/d)	60.8 (47.1, 77.0)	54.1 (41.6, 69.2)	67.2 (53.8, 83.1)	<0.001
Total carbohydrate intake (g/d)	232.7 (170.2, 306.8)	211.7 (149.6, 283.7)	252.4 (193.9, 323.9)	<0.001
Characteristics (*n* (%))				
Age group (year)				<0.001
18–44	4544 (35.5)	2167 (33.9)	2377 (37.1)	
45–59	4573 (35.7)	2266 (35.4)	2307 (36.0)	
≥60	3683 (28.8)	1967 (30.7)	1716 (26.8)	
Gender				0.017
Male	6025 (47.1)	2945 (46.0)	3080 (48.1)	
Female	6775 (52.9)	3455 (54.0)	3320 (51.9)	
BMI ^1^ (kg/m^2^)				<0.001
<24.0	6880 (55.4)	3619 (58.7)	3261 (52.2)	
24.0~27.9	4038 (32.5)	1882 (30.5)	2156 (34.5)	
≥28.0	1496 (12.1)	665 (10.8)	831 (13.3)	
Smoking status (%)				0.001
Non-smoker	8873 (69.3)	4395 (68.7)	4478 (70.0)	
Ex-smoker	555 (4.3)	242 (3.8)	313 (4.9)	
Current smoker	3367 (26.3)	1761 (27.5)	1606 (25.1)	
Unknown	5 (0.0)	2 (0.0)	3 (0.0)	
Drinking frequency (%)				<0.001
Never	8461 (66.1)	4451 (69.6)	4010 (62.7)	
Almost everyday	1275 (10.0)	604 (9.4)	671 (10.5)	
3–4 times per week	491 (3.8)	218 (3.4)	273 (4.3)	
1–2 times per week	951 (7.4)	416 (6.5)	535 (8.4)	
1–3 times per month	854 (6.7)	365 (5.7)	489 (7.6)	
<1 time per month	698 (5.5)	312 (4.9)	386 (6.0)	
Unknown	69 (0.5)	33 (0.5)	36 (0.6)	

The participants were divided into Q1 and Q2 by the median value of total sugar intake. Values are means ± standard errors (SEs), median (25th, 75th), or n (%). *p* values were calculated using Student’s *t*-test or the Mann–Whitney U test for continuous variables, and the chi-squared test for categorical variables. ^1^ CHNS: China Health and Nutrition Survey; SE: standard error; MET: metabolic equivalent; BMI: body mass index.

**Table 2:** The total and added sugar intake of diabetics and non-diabetics by age, gender, and BMI ^1^, CHNS ^1^ 2011.

		Total (*n* = 12,800)		Diabetes (*n* = 512)		Non-Diabetes (*n* = 12,288)		*p*-Value
		Mean ± SE ^1^	Median (25th, 75th)	Mean ± SE	Median (25th, 75th)	Mean ± SE	Median (25th, 75th)	
Age group (year)								
18–44	Total sugar intake (g/d)	29.7 ± 0.4	23.3 (11.3, 41.6)	25.3 ± 3.4	19.8 (13.0, 34.8)	29.8 ± 0.4	23.3 (11.2, 41.7)	0.630
	Total sugar (%E ^1^)	6.3 ± 0.1	5.3 (2.6, 8.7)	6.2 ± 0.8	5.4 (3.4, 7.4)	6.3 ± 0.1	5.3 (2.6, 8.7)	0.868
	Added sugar intake (g/d)	5.7 ± 0.2	0.3 (0.0, 5.0)	3.6 ± 1.1	0.5 (0.0, 6.5)	5.8 ± 0.2	0.3 (0.0, 5.0)	0.766
	Added sugar (%E)	1.2 ± 0.0	0.1 (0.0, 1.1)	0.8 ± 0.2	0.1 (0.0, 0.9)	1.2 ± 0.0	0.0 (0.0, 1.1)	0.098
45–59	Total sugar intake (g/d)	28.3 ± 0.4	22.2 (10.9, 39.1)	30.5 ± 1.8	25.3 (12.3, 43.6)	28.2 ± 0.4	22.1 (10.8, 39.0)	0.047
	Total sugar (%E)	5.9 ± 0.1	4.9 (2.4, 8.1)	6.2 ± 0.3	5.1 (2.9, 8.9)	5.9 ± 0.1	4.9 (2.3, 8.1)	0.060
	Added sugar intake (g/d)	4.9 ± 0.2	0.0 (0.0, 3.1)	5.2 ± 0.7	0.4 (0.0, 6.2)	4.9 ± 0.2	0.0 (0.0, 3.1)	0.008
	Added sugar (%E)	1.0 ± 0.0	0.0 (0.0, 0.7)	1.0 ± 0.1	0.1 (0.0, 1.3)	1.0 ± 0.0	0.0 (0.0, 0.7)	0.006
≥60	Total sugar intake (g/d)	26.1 ± 0.4	20.3 (9.6, 36.3)	28.6 ± 1.4	22.7 (13.2, 36.5)	25.9 ± 0.4	19.8 (9.4, 36.3)	0.004
	Total sugar (%E)	5.9 ± 0.1	4.9 (2.3, 8.3)	6.7 ± 0.3	6.1 (3.5, 8.9)	5.9 ± 0.1	4.8 (2.3, 8.2)	0.630
	Added sugar intake (g/d)	4.2 ± 0.2	0.0 (0.0, 2.5)	4.9 ± 0.9	0.3 (0.0, 3.1)	4.1 ± 0.2	0.0 (0.0, 2.4)	0.868
	Added sugar (%E)	0.9 ± 0.0	0.0 (0.0, 0.6)	1.0 ± 0.2	0.1 (0.0, 0.7)	0.9 ± 0.0	0.0 (0.0, 0.6)	0.766
Gender								
Male	Total sugar intake (g/d)	29.7 ± 0.3	22.6 (10.8, 41.4)	33.2 ± 1.7	28.1 (16.2, 46.0)	29.6 ± 0.3	22.5 (10.7, 41.2)	<0.001
	Total sugar (%E ^1^)	5.8 ± 0.1	4.8 (2.3, 8.0)	6.8 ± 0.3	5.8 (3.6, 9.2)	5.7 ± 0.1	4.7 (2.3, 7.9)	<0.001
	Added sugar intake (g/d)	6.2 ± 0.2	0.0 (0.0, 4.1)	6.3 ± 1.1	0.8 (0.0, 6.3)	6.2 ± 0.2	0.0 (0.0, 4.0)	0.005
	Added sugar (%E)	1.2 ± 0.0	0.0 (0.0, 0.8)	1.2 ± 0.2	0.2 (0.0, 1.6)	1.2 ± 0.0	0.0 (0.0, 0.8)	0.003
Female	Total sugar intake (g/d)	26.8 ± 0.3	21.4 (10.5, 37.2)	25.3 ± 1.3	20.7 (10.2, 32.8)	26.8 ± 0.3	21.4 (10.5, 37.4)	0.228
	Total sugar (%E)	6.3 ± 0.1	5.3 (2.6, 8.7)	6.2 ± 0.3	5.6 (2.7, 8.5)	6.3 ± 0.1	5.3 (2.6, 8.7)	0.754
	Added sugar intake (g/d)	3.9 ± 0.1	0.0 (0.0, 3.1)	3.6 ± 0.5	0.0 (0.0, 2.4)	3.9 ± 0.1	0.0 (0.0, 3.1)	0.800
	Added sugar (%E)	0.9 ± 0.0	0.0 (0.0, 0.7)	0.8 ± 0.1	0.0 (0.0, 0.7)	0.9 ± 0.0	0.0 (0.0, 0.7)	0.874
BMI (kg/m^2^)								
<24.0	Total sugar intake (g/d)	27.2 ± 0.3	20.6 (9.9, 37.4)	27.3 ± 1.5	20.2 (12.9, 38.4)	27.2 ± 0.3	20.6 (9.9, 37.4)	0.242
	Total sugar (%E ^1^)	5.9 ± 0.1	4.8 (2.3, 8.2)	6.4 ± 0.3	5.2 (3.4, 9.3)	5.9 ± 0.1	4.8 (2.3, 8.2)	0.011
	Added sugar intake (g/d)	4.8 ± 0.2	0.0 (0.0, 3.3)	3.9 ± 0.7	0.0 (0.0, 3.0)	4.8 ± 0.2	0.0 (0.0, 3.3)	0.746
	Added sugar (%E)	1.0 ± 0.0	0.0 (0.0, 0.7)	0.9 ± 0.1	0.0 (0.0, 0.8)	1.0 ± 0.0	0.0 (0.0, 0.7)	0.641
24.0–27.9	Total sugar intake (g/d)	29.5 ± 0.4	23.6 (11.8, 40.9)	31.6 ± 1.9	24.6 (14.4, 41.1)	29.4 ± 0.4	23.6 (11.7, 40.9)	0.290
	Total sugar (%E)	6.2 ± 0.1	5.3 (2.7, 8.6)	6.8 ± 0.3	6.1 (3.3, 8.8)	6.2 ± 0.1	5.3 (2.6, 8.6)	0.026
	Added sugar intake (g/d)	5.2 ± 0.2	0.2 (0.0, 3.9)	6.1 ± 1.2	0.8 (0.0, 5.6)	5.2 ± 0.2	0.1 (0.0, 3.7)	0.024
	Added sugar (%E)	1.0 ± 0.0	0.0 (0.0, 0.9)	1.2 ± 0.2	0.2 (0.0, 1.3)	1.0 ± 0.0	0.0 (0.0, 0.8)	0.016
≥28.0	Total sugar intake (g/d)	30.1 ± 0.6	25.0 (12.6, 41.4)	27.7 ± 1.9	23.0 (11.9, 37.8)	30.3 ± 0.7	25.3 (12.7, 41.5)	0.328
	Total sugar (%E)	6.5 ± 0.1	5.7 (2.9, 8.9)	6.3 ± 0.4	6.0 (2.9, 8.7)	6.5 ± 0.1	5.6 (2.9, 8.9)	0.967
	Added sugar intake (g/d)	5.3 ± 0.4	0.2 (0.0, 4.1)	4.1 ± 1.0	0.1 (0.0, 2.3)	5.4 ± 0.4	0.2 (0.0, 4.6)	0.348
	Added sugar (%E)	1.1 ± 0.1	0.0 (0.0, 1.0)	0.8 ± 0.2	0.0 (0.0, 0.6)	1.1 ± 0.1	0.0 (0.0, 1.0)	0.314

Values are means ± standard errors (SEs) and median (25th, 75th). *p* values were calculated using the Mann–Whitney U test. ^1^ BMI: body mass index; CHNS: China Health and Nutrition Survey; SE: standard error; %E: % of energy.

**Table 3:** The associations between the total and added sugar intake and diabetes status, CHNS ^1^ 2011.

Variable	Estimated Coefficient	SE ^1^	*p*-Value	OR ^1^ (95%CI ^1^)
Total sugar intake (per 1000 kcal)				
Model 1	0.010	0.004	0.004	1.010 (1.003, 1.017)
Model 2	0.008	0.004	0.022	1.008 (1.001, 1.016)
Added sugar intake (per 1000 kcal)				
Model 1	0.004	0.007	0.525	1.004 (0.991, 1.018)
Model 2	0.004	0.007	0.601	1.004 (0.990, 1.018)

OR represents the multiple increases in the odds of diabetes associated with 1000 kcal in the total or added sugar intake. Model 1: adjusted for age and gender; Model 2: adjusted for age, gender, smoking status, drinking frequency, and physical activity. ^1^ CHNS: China Health and Nutrition Survey; SE: standard error; OR: odds ratio; CI: confidence interval; BMI: body mass index.

**Table 4:** The associations between sugar intake and diabetes status by the BMI, CHNS ^1^ 2011.

Variable	B	SE ^1^	*p*-Value	OR ^1^ (95%CI ^1^)
BMI < 24.0 kg/m^2^				
Total sugar intake (per 1000 kcal)				
Model 1	0.013	0.006	0.025	1.013 (1.002, 1.025)
Model 2	0.012	0.006	<0.05 *	1.012 (1.000, 1.024)
Added sugar intake (per 1000 kcal)				
Model 1	−0.002	0.013	0.885	0.998 (0.973, 1.024)
Model 2	−0.002	0.014	0.904	0.998 (0.972, 1.025)
24.0 kg/m^2^ ≤ BMI < 28.0 kg/m^2^				
Total sugar intake (per 1000 kcal)				
Model 1	0.009	0.005	0.102	1.009 (0.998, 1.020)
Model 2	0.008	0.006	0.151	1.008 (0.997, 1.019)
Added sugar intake (per 1000 kcal)				
Model 1	0.010	0.010	0.274	1.010 (0.992, 1.029)
Model 2	0.010	0.010	0.323	1.010 (0.991, 1.029)
BMI > 28.0 kg/m^2^				
Total sugar intake (per 1000 kcal)				
Model 1	−0.004	0.009	0.680	0.996 (0.978, 1.014)
Model 2	−0.007	0.009	0.468	0.993 (0.975, 1.012)
Added sugar intake (per 1000 kcal)				
Model 1	−0.013	0.022	0.561	0.987 (0.946, 1.030)
Model 2	−0.017	0.022	0.429	0.983 (0.941, 1.026)

OR represents the multiple increases in the odds of diabetes associated with 1000 kcal in the total or added sugar intake. Model 1: adjusted for age and gender; Model 2: adjusted for age, gender, smoking status, drinking frequency, and physical activity. * *p* value = 0.04995. ^1^ CHNS: China Health and Nutrition Survey; SE: standard error; OR: odds ratio; CI: confidence interval; BMI: body mass index.

## Data Availability

Publicly available datasets were analyzed in this study. This data can be found here: [https://www.cpc.unc.edu/projects/china/data].
